# The Present State of Existential Interventions Within Palliative Care

**DOI:** 10.3389/fpsyt.2021.811612

**Published:** 2022-01-13

**Authors:** Takeshi Terao, Moriaki Satoh

**Affiliations:** Department of Neuropsychiatry, Oita University Faculty of Medicine, Oita, Japan

**Keywords:** existentialism, existential psychotherapy, advanced cancer, palliative care, terminal care, meaning, existential interventions

## Abstract

Existential psychotherapy is rooted in the European tradition of existential philosophy. Existential philosophers include Husserl and Heidegger, who were German, and Camus, Sartre, de Beauvoir, and Merleau-Ponty, who were French. Their works contain existentially ultimate themes such as death, freedom, meaninglessness, and isolation. Based on their knowledge of existential philosophy, Binswanger, Frankl, and Boss developed the earlier existential psychotherapies such as Dasein-analysis and Logotherapy, while May, Laing, Yalom, May, and Wong started later existential psychotherapies in the British and American culture. Focusing on patients with advanced cancer and/or terminal care, we found nine types of existential psychotherapies which were investigated using randomized controlled trials (RCTs): Meaning-Centered Group Psychotherapy (MCGP), Individual Meaning-Centered Psychotherapy (IMCP), Meaning-Making intervention (MMi), Meaning of Life Intervention, Managing Cancer and Living Meaningfully (CALM), Hope Intervention, Cognitive and Existential Intervention, Dignity Therapy, and Life-Review Interviews, from 19 relevant RCTs. All deal with death, meaninglessness, isolation, and freedom. Particularly, MCGP, IMCP, MMi, Meaning of Life intervention, and CALM emphasize finding and/or making meaning in the individual's life. The effects on existential or spiritual well-being were confirmed in MCGP, IMCP, Meaning of Life intervention, and Life-Review intervention although the number of studies were very few. In the other interventions, there were heterogenous findings and again the number of studies was very small. Further studies are required to investigate the effects of existential psychotherapy on patients with advanced cancer.

## Introduction

Yalom (1) defined existential psychotherapy as a dynamic approach to therapy which focuses on ultimate concerns such as death, freedom, isolation, and meaninglessness. According to Yalom (1), death is the most obvious and the most easily apprehended ultimate concern, where core existential conflict is the tension between the awareness of the inevitability of death and the wish to continue to be. Freedom refers to the absence of external structure where the individual is entirely responsible for his or her own world, life design, choices, and actions. Isolation is intrapersonal and fundamental, one where the existential conflict is the tension between our awareness of our absolute isolation and our wish for contact, protection, and belongingness to a larger whole. Meaninglessness is the existential dynamic conflict stemming from the dilemma of a meaning-seeking creature who is thrown into a universe that has no meaning.

According to the review by Balogh et al. (2), it was Edmund Husserl, a German philosopher, who first gave the term phenomenology its philosophical characteristics and nuanced meaning. However, it was Martin Heidegger, originally an assistant of Husserl, who critically transformed Husserl's insights in his famous 1927 work “Being and Time,” and carried them over to the spheres of ontology, which is a philosophical field that deals with the existence of all beings. Heidegger coined the term “Dasein” as existential possibilities and limitations and initiated a prominent philosophical and literary movement that came to be called “existentialism.” Jean-Paul Sartre, Albert Camus, Simone de Beauvoir, Karl Jaspers, Maurice Merleau-Ponty, Arthur Schopenhauer, Soren Kierkegaard, Fyodor Dostoevsky, and Friedrich Nietzsche are all well-known existentialists.

Existential psychotherapy is a unique approach in that it scrutinizes psychic phenomena from an existentialist point of view. In the beginning, Ludwig Binswanger, and Medard Boss had attempted to elaborate Heidegger's ontology to make it practical in clinical settings. Around the same time Austrian-born Viktor Frankl also established Logotherapy, which aims to help clients establish meaning and purpose in their lives. By the end of the 1950's, the existential approach to psychiatry and psychotherapy arrived and started to grow in the US. Along with the so-called humanistic movement in psychology, it has gained considerable influence after a relatively brief period. Rollo May and Irvin D. Yalom are the most well-known and acknowledged representatives of the American school of existential therapy. Existential psychotherapy is a rich tradition all over the world, with more than 120 existential psychotherapy institutions, and existential psychoterapy is used for a broad range of problems (3–5).

The present century may be called an age of anxiety because of the increase in existential anxiety due to Covid-19, climate change and the digital revolution. Therefore, existential therapy is much needed for all people. Indeed, existential therapy is much broader and it is applicable to all kinds of clients (6–9). Leading theorists and writers within existential psychotherapy in the last two decades, are Emily van Deurzen and Kirk J. Schneider who mainly write theoretical and clinical papers and Mick Cooper who is also an empirical researcher. Their clinical scope is much wider than expected. Nonetheless, existential interventions have proven most effective for patients with serious and life-threatening illnesses, as pointed out in the meta-analyisis (10), which showed that research on existential interventions is quite limited, and more systematic research on the effectiveness of existential interventions for other groups of patients are needed and that although evidence seems strongest for serious ill patients, present application of existential psychotherapy is not limited to this group. Unfortunately, there has not been a strong tradition for research within existential psychotherapy. Some therapists use existential therapy as an overarching framework, and integrate interventions from other and evidence-based approaches (11), and there is a huge overlap between existential and other humanistic-experiential approaches, that have a stronger evidence base (12).

According to Wong et al. (13), the worst of times can become the best of times for any individual with the necessary inner resources such as meaning, faith, courage, and creativity, and the mission of existential positive psychology is to investigate ways to reduce human suffering and transform it into human flourishing. Existential positive psychology represents a unique kind of second wave positive psychology (PP2.0) because it embraces the human complexity of existentialism and Taoism. PP2.0 goes beyond mere recognition of polarity, and it makes the bold assumptions that (a) suffering is necessary for flourishing and (b) that enduring happiness and well-being can only be achieved through the dialectical integration of opposites. Moreover, Wong et al. (13) supported the thesis that suffering triggers the search for meaning, or self-transcendence, which in turn function as a buffer against the adverse effects of suffering. All the good things we value and cherish are on the other side of suffering; we will not be able to fulfill our dreams without a resilient mindset to embrace sacrifices and to go through the gates of fears and suffering. Sustainable well-being can be achieved through learning how to make the best use of the dynamic and dialectic interplay between positive and negative life experiences in each context (13). This PP 2.0, as conceptualized by Wong, proposed that the most promising strategy to accomplish the mission of positive psychology is to confront the dark side of human existence and understand the unique experience and expression of well-being in different cultures. Thus, PP 2.0 emphasized the existential universal on one hand, and indigenous cultural expression on the other hand. Specifically, PP2.0 introduces the following principles and practices: (1) Accepting and confronting with courage the reality that life is full of evil and suffering; (2) sustainable well-being can only be achieved through overcoming suffering and the dark side of life; (3) recognizing that everything in life comes in polarities and the importance of achieving an adaptive balance through dialectics; (4) learning from indigenous psychology, such as the ancient wisdom of finding deep joy in bad situations (14). In contrast to the dominant message of American positive psychology that emphasizes what is good and right in life, the message of PP 2.0 and meaning therapy is that life is full of suffering and injustice. We need to give up our unrealistic expectations of only good things and confront life with a sense of tragic optimism and struggle for a better future with courage (15).

In this review, we focused on the papers dealing with existential psychotherapy for patients with advanced cancer which were published around the last two decades to identify the present state of existential interventions within pallitative care.

## Materials and Methods

This review was qualitative and not systematic one. It was conducted in October 2021. We searched the PubMed database using search words “existential therapy,” “existential approach,” or “existential intervention,” and “advanced cancer,” “terminal care,” or “palliative care,” augmented by bibliographic cross-referencing to identify relevant articles. Moreover, inclusion criterion was randomized controlled trial (RCT). We compared the content of interventions, specifically their ultimate concerns (i.e., death, freedom, isolation, and/or meaninglessness), and their effects on existential or spiritual well-being. Spiritual well-being consists of not only a religious component, which refers to one's faith, but also an existential component, which refers to one's sense of meaning and purpose in life (16). Although most studies have investigated improvements in psychiatric symptoms such as depression and anxiety, these seem to be rather non-specific changes due to existential psychotherapy; thus, we did not attach importance to them in this review.

## Results

We found nineteen RCTs, most of which included patients with advanced cancer. We introduced the content of each type of psychotherapy briefly and reviewed the effects on existential or spiritual well-being.

### Meaning-Centered Group Psychotherapy

According to Breitbart et al. (17), they developed Meaning-Centered Group Psychotherapy (MCGP) to help patients with advanced cancer sustain or enhance a sense of meaning, peace, and purpose in their lives even as they approach the end of life. This manualized 8-week intervention, which is influenced by the work of Viktor Frankl, utilizes didactics, discussion, and experiential exercises that focus on themes related to meaning and advanced cancer. Each session addresses specific themes related to an exploration of the concepts and sources of meaning, the relationship and impact of cancer to one's sense of meaning and identity, and placement of one's life in a historical and personal context (i.e., understanding one's “legacy”). With regard to the content of intervention, MCGP deals with meaning (for mitigating meaninglessness), death (because of cancer), and isolation (relationships with the outer world), and freedom.

Breitbart et al. (17) performed an RCT where 90 patients with advanced cancer were randomly assigned to either MCGP or a supportive group psychotherapy (SGP). Results have shown that MCGP resulted in significantly greater improvements in spiritual well-being than SGP.

Breitbart et al. (18) further performed an RCT where 253 patients with advanced cancer were randomly assigned to MCGP or SGP. As a result, MCGP significantly increased spiritual well-being than SGP.

### Individual Meaning-Centered Psychotherapy

Breitbart et al. (19) developed Individual Meaning-Centered Psychotherapy (IMCP) to address the need for brief interventions targeting spiritual well-being and meaning for patients with advanced cancer. Because of the inflexibility inherent in group interventions, they adapted MCGP to an individual intervention in hopes of reducing attrition and missed sessions while maintaining the benefits. IMCP is a manualized 7-week intervention designed to assist patients with advanced cancer in sustaining or enhancing a sense of meaning, peace, and purpose in their lives as they face limitations due to the progression of disease and treatment. Similar to MCGP, IMCP deals with meaning (for mitigating meaninglessness), death (because of cancer), and isolation (relationships with the outer world), and freedom.

Breitbart et al. (19) performed an RCT where 120 patients with advanced cancer were randomly assigned to IMCP or therapeutic massage. As a result, IMCP significantly improved spiritual well-being compared to therapeutic massage.

Breitbart et al. (20) further performed an RCT, where 321 patients with advanced cancer were randomly assigned to IMCP, supportive psychotherapy, or enhanced usual care. As a result, MCGP significantly increased spiritual well-being than enhanced usual care.

### Meaning-Making Intervention

According to Lee et al. (21), meaning-making intervention (Mmi) was originally developed to help patients with post-traumatic distress symptoms and their families. It was further modified for cancer patients since the cancer experience shares many of the features of traumatic injury, such as persistent re-experiencing of the stressful event in the form of flashbacks or nightmares. Overall, patients participated in three to eight sessions on a daily, weekly, or monthly basis that ranged from ten minutes to 3 h in length, depending on each patient's needs and states. There were three tasks: Task 1 Acknowledge the present (i.e., to provide a secure context to revisit events since the cancer diagnosis, telling his or her story, understanding what happened to the self, and grieving for losses); Task 2, Contemplate the past (i.e., to embed the new cancer experience within a familiar framework of past challenges, reflection on one's life and reflecting on how past challenges were overcome); and Task 3, Commit to the present for the future (i.e., to reestablish a sense of commitment toward meeting attainable goals in the context of one's mortality and acknowledging one's mortality). MMi deals with meaning (for mitigating meaninglessness), death (because of cancer) and isolation (relationships with the outer world), and freedom.

Lee et al. (22) performed an RCT where 82 breast or colorectal cancer patients were randomly assigned to MMi or routine care (i.e., control group). As a result, the experimental group participants demonstrated significantly higher levels of self-esteem, optimism, and self-efficacy compared to the control group after controlling for baseline scores. This study did not measure existential or spiritual well-being. However, MMi significantly increased self-esteem, optimism, and self-efficacy than control group, which may be associated with existential or spiritual well-being.

Henry et al. (23) performed an RCT where 24 patients with advanced cancer were randomly allocated to either an MMi group or control group. The results showed that existential well-being was not significantly different between the MMi group and control group.

### Meaning of Life Intervention

Mok et al. (24) developed a brief intervention that would help participants reflect on their lives based on the sources of meaning of life proposed in logotherapy (i.e., creative, experiential, and attitudinal values). The first question, “What do you think about your life?,” facilitates a review of significant life events, primarily creative values. The second question, “How have you faced adversities in life?,” aims to explore internal and external resources, primarily attitudinal values. The third, fourth, and fifth questions “What do you do to love yourself and others?;” “What brings you joy?;” and “What do you appreciate in your life?” aimed to facilitate a review of relationships with the outside world, primarily experiential values. This intervention deals with meaning (for mitigating meaninglessness), death (because of cancer), and isolation (relationships with the outer world), and freedom.

Mok et al. (24) performed an RCT where 84 patients with advanced cancer were randomly allocated to either the Meaning of Life intervention group or the control group. The results showed that existential distress significantly improved in the intervention group, when compared to control group.

### Managing Cancer and Living Meaningfully (CALM)

Rodin et al. (25) developed a novel, brief, and tailored supportive expressive psychotherapeutic intervention, referred to as Managing Cancer and Living Meaningfully (CALM) for patients with advanced cancer and a prognosis of at least 1 year to live. On the basis of relational, attachment, and existential theory, CALM provides a therapeutic relationship and reflective space, with attention to the following domains: symptom management and communication with health care providers, changes in self and relations with close others, spiritual well-being and the sense of meaning and purpose, and mortality and future-oriented concerns. CALM deals with meaning (for mitigating meaninglessness), death (because of cancer), and isolation (relationships with the outer world), and freedom.

Rodin et al. (25) performed an RCT where 305 patients with advanced cancer were randomly assigned to either CALM or usual care. As a result, spiritual well-being was not significantly different between CALM and usual care.

### Hope Intervention

Herth (26) defined hope as a multidimensional and dynamic life force characterized by a confident yet uncertain expectation of achieving good. Hope intervention consists of four elements: Searching for hope (experiential process), Connecting with others (relational process), Expanding the boundaries (spiritual/transcendent process), and Building the hopeful veneer (rational thought process). This intervention deals with meaning (as a basis of one's hope), death (because of cancer), and isolation (relationships with the outer world), and freedom.

Herth (26) performed an RCT, where 115 people with a first recurrence of cancer were randomly assigned to one of three groups: treatment group (i.e., hope), attention control group (i.e., informational), or control group (i.e., usual treatment). This study did not measure existential or spiritual well-being. However, the treatment group demonstrated significantly increased hope than control groups, which may be associated with existential or spiritual well-being.

Duggleby et al. (27) performed an RCT where 60 terminally ill cancer patients who were randomly assigned to an intervention group or control group. As a result, a subscale of “existential” was significantly better in the intervention group than in the control group.

### Cognitive-Existential Intervention

According to Kissane et al. (28), this intervention typically began with a series of patient's narratives of their experience of illness. Early phase focused on grief and existential concerns, while cognitive aspects were integrated during the middle phase. Typical themes included the threat of death; fear of recurrence; living with uncertainty; understanding treatment with chemotherapy, radiotherapy and hormones; the doctor–patient relationship; body- and self-image; sexuality; surgical reconstruction; relationship with partner, friends and family; and lifestyle and future goals. This intervention deals with death (because of cancer), isolation (relationships with the outer world) and meaning (related with treatment), and freedom.

Kissane et al. (28) performed an RCT where 303 women with early-stage breast cancer were randomly assigned to either a group that received 20 weekly group therapy sessions in addition to three relaxation classes or to a control group receiving only three relaxation classes. This study did not measure existential or spiritual well-being.

Gagnon et al. (29) performed an RCT where 33 non-metastatic cancer patients were randomized between the group intervention, the individual intervention, and the usual condition of care. The results showed that existential quality of life was not significantly different across the three groups.

### Dignity Therapy

Chochinov et al. (30) invented Dignity Therapy based on their dignity model of palliative care, which has seven dignity themes such as generativity, continuity of self, role preservation, maintenance of pride, hopefulness, aftermath concerns, and care tenor. This intervention deals with meaning (as a basis of one's dignity), death (because of cancer) and isolation (relationships with the outer world), and freedom.

Chochinov et al. (31) conducted the first RCT where patients receiving hospital or community-based (i.e., hospice or home) palliative care, were randomly assigned to Dignity Therapy, Client Centered Care, or Standard Palliative Care. The results of the study showed that there were no significant differences across three groups in changes of the functional assessment of chronic illness therapy-spiritual well-being scale scores. However, conflicting findings show that Dignity Therapy significantly improved the spiritual well-being as compared to Client-Centered Care in Post Study Survey.

Hall et al. (32) performed an RCT where 45 patients with advanced cancer were randomly allocated to receive either an intervention plus standard care or standard care only (i.e., control group). This study did not measure existential or spiritual well-being. However, findings show that Dignity Therapy did not improve dignity-related distress, which may be associated with issues of existential or spiritual well-being.

Julião et al. (33) performed an RCT where 80 patients with terminal illness were randomly assigned to an intervention group that received Dignity Therapy and standard palliative care or to a control group that received standard palliative care only. This study did not measure existential or spiritual well-being.

Julião et al. (34) reanalyzed the data of the previous RCT (33). The main outcomes were demoralization syndrome, the desire for death, and a sense of dignity. This study did not measure existential or spiritual well-being. The results show that Dignity Therapy was associated with a significant decrease in desire for death as compared with standard palliative care, but was not significantly associated with a decrease in desire for death. Although existential or spiritual well-being may be negatively associated with demoralized syndrome and desire for death, the findings were conflictive.

Vuksanovic et al. (35) performed an RCT where 70 adults with advanced terminal disease were randomly assigned to Dignity Therapy, life review, and waitlist control. As a result, Dignity Therapy demonstrated significantly increased generativity and ego-integrity as compared to LR and WC group's scores at study completion. Although this study did not measure existential or spiritual well-being, existential or spiritual well-being may be positively associated with generativity and ego-integrity.

### Life-Review Interviews

According to Ando et al. (36), they developed Short-Term Life Review to have two interview sessions. In the first session, the patient reviewed his or her life with an interviewer who was trained to conduct the therapy. Each interview session lasted 30 to 60 min, with a one-week interval between the first and second sessions. This intervention deals with meaning (from a life review), death (because of cancer) and isolation (relationships with the outer world), and freedom.

Ando et al. (36) performed an RCT where 68 terminally ill cancer patients were randomly assigned to either the Short-Term Life-Review interview group or the control group. As a result, this intervention significantly improved spiritual well-being as compared to the control.

Xiao et al. (37) performed an RCT where 80 patients with advanced cancer were randomly assigned to Life review program group or control group. As a result, this intervention significantly improved existential distress than control.

## Discussion

In the present review, we investigated nine types of existential psychotherapies. As shown in Table 1, MCGP, IMCP, MMi, Meaning of Life intervention, and CALM are considered to explicitly deal with meaninglessness as one of existentially ultimate concerns and their purposes may be to find and/or make meaning in the individual's life. In Dignity therapy, meaning may be a basis of dignity while in Life-review interventions, life itself may have the meaningful story. Cognitive and existential interventions may also be associated with meaning as patients may find their meaning through the course of intervention. Meanwhile, hope itself may be associated with meaning in the near future. All interventions may deal with death (because the patients had advanced cancer), isolation (because the patients tended to lose the connection with the outer world) and freedom.

**Table 1:** Existential psychotherapy for existentially ultimate concerns and existential or spiritual well-being.

	**Death**	**Meaninglessness**	**Isolation**	**Freedom**	**Effects on existential or spiritual well-being**
MCGP	○	⊚	○	○	Two RCTs confirmed the effects.
IMCP	○	⊚	○	○	Two RCTs confirmed the effects.
MMi	○	⊚	○	○	One RCT was N/A and another RCT did not confirm the effects.
Meaning of Life	○	⊚	○	○	One RCT confirmed the effects.
CALM	○	⊚	○	○	One RCT did not confirm the effects.
Hope	○	○	○	○	One was N/A and another confirmed the effects.
Cognitive and Existential	○	○	○	○	One RCT was N/A and another did not confirm the effects.
Dignity	○	○	○	○	One showed conflicting findings and four RCTs were N/A.
Life-Review	○	○	○	○	Two RCTs confirmed the effects.

*MCGP, Meaning-Centered Group Psychotherapy; IMCP, Individual Meaning-Centered Psychotherapy; Mmi, Meaning-Making intervention; CALM, Managing Cancer and Living Meaningfully; ○ means the presence of intervention in the existentially ultimate concern while ⊚ means the prominent intervention in it*.

With regard to freedom, for patients with advanced cancer, there is a freedom to select life or death, treatment or non-treatment, home-based care or hospital-based care. Therefore, we consider that all interventions may deal with freedom implicitly or explicitly. Therefore, all nine types of existential psychotherapies deal with death, meaninglessness, isolation, and freedom. MCGP, IMCP, MMi, Meaning of Life intervention, and CALM particularly emphasize finding and/or making meaning in the individual's life. Also shown in Table 1, the effects on existential or spiritual well-being were confirmed in MCGP, IMCP, Meaning of Life intervention, and Life-Review intervention, though the number of studies were very small. There were heterogenous findings in other interventions, however, the number of studies were very few. Therefore, the effects on existential or spiritual well-being by existential psychotherapy in patients with advanced cancer are yet to be determined. For reference, Bauereiß et al. (16) showed the effects on existential well-being in Hedges g were 1.39 (95% CI: 1.02–1.75) in Life Review, 0.58 (0.05–1.11) in Hope intervention, 0.39 (0.11–0.66) in Meaning-Centered Psychotherapy, and 0.07 (-0.17–0.30) in Dignity therapy. Those findings may agree with our findings (Table 1).

Grossman et al. (38) performed a systematic review of death anxiety intervention in patients with advanced cancer. The results of the study showed that Life Review (36) brought about a significant improvement in existential suffering, whereas Chochinov's large multinational RCT on Dignity Therapy yielded no significant improvement in existential domains. Strictly speaking, there were no significant differences across three groups in changes of the functional assessment of Chronic Illness Therapy-Spiritual Well-Being scale scores, but Dignity Therapy significantly improved spiritual well-being as compared with Client Centered Care in a post-study survey. These are conflicting findings and are therefore not robust. Surprisingly, none of the five RCTs could confirm the effects of Dignity Therapy on existential or spiritual well-being. Although the reason for the lack of effects is unclear, we wonder whether dignity itself is important for existential or spiritual well-being. With regard to PP2.0, we could not find RCTs on patients with advanced and/or terminal stage cancer, but hopefully new RCTs will be planned to investigate the effects of PP2.0 for patients with advanced cancer because the primary endpoint of our review was existential or spiritual well-being, and wellbeing can be conceptualized in PP2.0 from a golden triangle where each of the three components of this triad (faith, meaning, and relationship) is possible due to the of double helix of self-detachment (mindfulness) and self-transcendence (meaning) that liberate us from self-absorption (15). Recently, we developed Existential and Mindfulness–Based Intervention (EXMIND) in which we performed a life-scan where individuals were instructed to recall past successful (reasonable) experiences and their success was commended by actual working members, which probably brought about meaningfulness and pleasure of achieving purpose. Also, individuals were asked to recall past miserable (unreasonable) experiences, and their patience and perseverance were respected by the actual working members, which probably actualized self-love (or self-esteem). Moreover, we encouraged them to think about the purpose and meaning of life not attributed to common sense but to their proper thought, to accept themselves as they are, to understand that they are happy when they think so during individual interviews, and to confirm individual will to live better in the uncertain future during individual interviews (39–41). Similar to PP2.0 which adds depth to counseling psychology by applying the paradoxical principle of treating suffering as the foundation for sustainable well-being, our EXMIND also deals with the dark side of life (unreasonable unsuccessful experiences) and the sunny side of life (reasonable successful experiences), which may be well applied also to patients with advanced cancer. In any case, further studies are required to investigate such effects using RCTs.

In conclusion, there have been various types of existential psychotherapies, all of which deal with death, meaninglessness, isolation, and freedom. Moreover, the effects on existential or spiritual well-being were confirmed in several psychotherapies, but the number of studies were very few. Further studies are required to investigate the effects of existential interventions on existential or spiritual well-being within palliative care.

## Author Contributions

TT had an idea and wrote the manuscript. MS read and commented it constructively. Both rewrote the manuscript and approved of the final version.

## Conflict of Interest

The authors declare that the research was conducted in the absence of any commercial or financial relationships that could be construed as a potential conflict of interest.

## Publisher's Note

All claims expressed in this article are solely those of the authors and do not necessarily represent those of their affiliated organizations, or those of the publisher, the editors and the reviewers. Any product that may be evaluated in this article, or claim that may be made by its manufacturer, is not guaranteed or endorsed by the publisher.
